# Learning to Crawl: Determining the Role of Genetic Abnormalities on Postoperative Outcomes in Congenital Heart Disease

**DOI:** 10.1161/JAHA.122.026369

**Published:** 2022-09-29

**Authors:** Benjamin J. Landis, Benjamin M. Helm, Jeremy L. Herrmann, Madeline C. Hoover, Matthew D. Durbin, Lindsey R. Elmore, Manyan Huang, Michael Johansen, Ming Li, Leon F. Przybylowski, Gabrielle C. Geddes, Stephanie M. Ware

**Affiliations:** ^1^ Division of Pediatric Cardiology, Department of Pediatrics, Riley Hospital for Children Indiana University School of Medicine Indianapolis IN; ^2^ Department of Medical and Molecular Genetics Indiana University School of Medicine Indianapolis IN; ^3^ Division of Thoracic and Cardiovascular Surgery Indiana University School of Medicine Indianapolis IN; ^4^ Division of Neonatal‐Perinatal Medicine, Riley Hospital for Children Indiana University School of Medicine Indianapolis IN; ^5^ Department of Pediatrics Indiana University School of Medicine Indianapolis IN; ^6^ Department of Epidemiology and Biostatistics Indiana University Bloomington School of Public Health Bloomington IN

**Keywords:** cardiothoracic surgery, chromosomal microarray, congenital heart disease, copy‐number variants, genetics, Clinical Studies, Genetics, Mortality/Survival

## Abstract

**Background:**

Our cardiac center established a systematic approach for inpatient cardiovascular genetics evaluations of infants with congenital heart disease, including routine chromosomal microarray (CMA) testing. This provides a new opportunity to investigate correlation between genetic abnormalities and postoperative course.

**Methods and Results:**

Infants who underwent congenital heart disease surgery as neonates (aged ≤28 days) from 2015 to 2020 were identified. Cases with trisomy 21 or 18 were excluded. Diagnostic genetic results or CMA with variant of uncertain significance were considered abnormal. We compared postoperative outcomes following initial congenital heart disease surgery in patients found to have genetic abnormality to those who had negative CMA. Among 355 eligible patients, genetics consultations or CMA were completed in 88%. A genetic abnormality was identified in 73 patients (21%), whereas 221 had negative CMA results. Genetic abnormality was associated with prematurity, extracardiac anomaly, and lower weight at surgery. Operative mortality rate was 9.6% in patients with a genetic abnormality versus 4.1% in patients without an identified genetic abnormality (*P*=0.080). Mortality was similar when genetic evaluations were diagnostic (9.3%) or identified a variant of uncertain significance on CMA (10.0%). Among 14 patients with 22q11.2 deletion, the 2 mortality cases had additional CMA findings. In patients without extracardiac anomaly, genetic abnormality was independently associated with increased mortality (*P*=0.019). CMA abnormality was not associated with postoperative length of hospitalization, extracorporeal membrane oxygenation, or >7 days to initial extubation.

**Conclusions:**

Routine genetic evaluations and CMA may help to stratify mortality risk in severe congenital heart disease with syndromic or nonsyndromic presentations.

**Nonstandard Abbreviations and Acronyms**
CMA: chromosomal microarray
CNV: copy‐number variant
CPB: cardiopulmonary bypass
CVG: cardiovascular genetics
ECA: extracardiac anomaly
STAT: Society of Thoracic Surgeons‐European Association for Cardio‐Thoracic Surgery
STS: Society of Thoracic Surgeons
VUS: variant of uncertain significance

**Clinical Perspective**

**What Is New?**

- The routine application of formal inpatient cardiovascular genetics evaluations for neonates undergoing congenital heart disease surgery facilitated analysis of early postoperative outcomes in a genetically well‐defined clinical population.
- Genetic abnormalities that likely would not have been otherwise detected were associated with mortality in patients with syndromic or nonsyndromic presentations.

**What Are the Clinical Implications?**

- More widespread adoption of routine genetic evaluation practices in neonates with severe congenital heart disease will be critical to define the clinical impact of genetic abnormalities and foster tailored care that improves outcomes.

Congenital heart disease (CHD) causes significant infant morbidity and mortality.^1^ Chromosomal microarray (CMA) is a genome‐wide test that identifies copy‐number variants (CNVs) including deletions or duplications or regions of homozygosity. CMA can identify genetic syndromes that have high penetrance of CHD, such as 22q11.2 deletion and 7q11.23 deletion (Williams syndrome). CMA can also identify more rare genomic disorders with CHD association such as 8p23.1 duplication, 16p11.2 deletion, or 15q11.2 (BP1‐BP2) deletion.^2^, ^3^, ^4^ Pathogenic CNVs have been reported in patients with extracardiac anomalies (ECAs) as well as in those with isolated CHD.^5^ As we have recently described in detail, in 2014 the cardiovascular genetics (CVG) program at Riley Hospital for Children at Indiana University Health implemented a clinical algorithm for the genetic evaluation of infants hospitalized with CHD, which included routine CMA testing of patients with any class of severe CHD.^6^

Prior literature has suggested, to varying degrees, that genetic diagnoses are associated with worse postoperative outcomes in infants.^7^, ^8^ The understanding of CNVs that cause CHD has advanced rapidly, and the routine clinical application of CMA has increased identification of CNVs in this population. However, the use of CMA is still variable between different pediatric cardiac centers. Therefore, current understanding of the impact of CNVs on surgical outcomes in the era of clinical CMA testing is incomplete and unable to be ascertained from current multi‐institutional surgical outcomes databases. Our standardized and routine performance of CVG evaluations at our center provides a new opportunity to understand these risks. Therefore, the objective of this study was to determine the impact of genetic abnormalities including CNVs on early postoperative outcomes following neonatal CHD surgery.

## METHODS

### Transparency and Openness

The data that support the findings of this study are available from the corresponding author upon reasonable request.

### Study Population and Data Collection

Infants undergoing cardiac surgery for CHD from January 1, 2015 to March 1, 2020 at age ≤28 days were identified through the Society of Thoracic Surgeons (STS) Congenital Heart Surgery Database at Indiana University Health. Cardiac transplants and patent ductus arteriosus ligations were excluded. Each patient's first cardiac surgery entered into the database was selected for analysis. Demographic and clinical data were primarily collected from STS data. Data entered into the noncardiac congenital anatomic abnormalities field of the STS database were included as ECAs. Intubation prior to CHD surgery was defined as intubation that occurred >24 hours before surgery. The STS‐European Association for Cardio‐Thoracic Surgery (STAT) mortality risk scores (range from 1 to 5) were designated per STS protocol.^9^ The STS defined operative mortality as death that occurred during the same hospitalization or within 30 days of the operation. The number of days of intubation was calculated from the time of operation to initial extubation. Patients who died before postoperative day 8 were excluded from extubation analysis. Patients who died before hospital discharge were not included in the analysis of length of postoperative hospitalization.

### Genetics Evaluations

The records of genetic testing completion, results, and interpretation were collected from the electronic medical record along with information from the genetics consultation, if performed. Clinical genetic testing was performed in clinical laboratories using standard methods or in‐house as previously described.^6^ CMA was performed routinely per the clinical algorithm. The indications for additional molecular gene testing were determined by the consulting clinical geneticist. Geneticists and genetic counselors reviewed all testing reports, including clinical interpretation of CNVs, as part of standard inpatient consultation practices. CMA and molecular genetic testing results were classified as (1) normal, (2) variants of uncertain significance (VUSs), or (3) diagnostic (ie, pathogenic or likely pathogenic) according to established guidelines.^10^, ^11^ For this study, the definition of genetic abnormality was (1) CMA abnormality, including CNVs classified as diagnostic or VUS or (2) genetic syndrome identified by a geneticist clinically or with additional genetic testing at the time of initial consult. Parental testing for CNVs was pursued when clinically indicated. ROHs as the only CMA finding did not qualify patients as having genetic abnormality. Patients with trisomy 21 (N=6) or trisomy 18 (N=1) were excluded from the study.

### Statistical Analysis

Categorical variables are reported as frequency and percent. Variance in continuous variables is reported as standard deviation. Univariate analyses of patients' characteristics and postoperative outcomes included Pearson χ^2^ test (all expected cell counts in contingency table ≥5) or Fisher exact test (any expected cell counts <5) for categorical variables and 2‐sample *t* test for continuous variables. The effect of genetic abnormality on postoperative outcomes was estimated by multivariate linear or logistic regression, adjusting for cardiopulmonary bypass (CPB) status, prematurity, sex, weight at CHD surgery, intubation status before CHD surgery, and STAT mortality risk category. Statistical analyses were performed in R version 4.0.2 (R Foundation for Statistical Computing). The corresponding author (B.J.L.) had full access to all data in the study and takes responsibility for its integrity and the data analysis.

### Human Subjects

The study was approved by the institutional review board at Indiana University (Protocol ID: 1408953015) and used a waiver of informed consent. Study procedures followed were in accordance with institutional guidelines.

## RESULTS

### Rates of Genetic Evaluations and Yields

**Figure 1:**
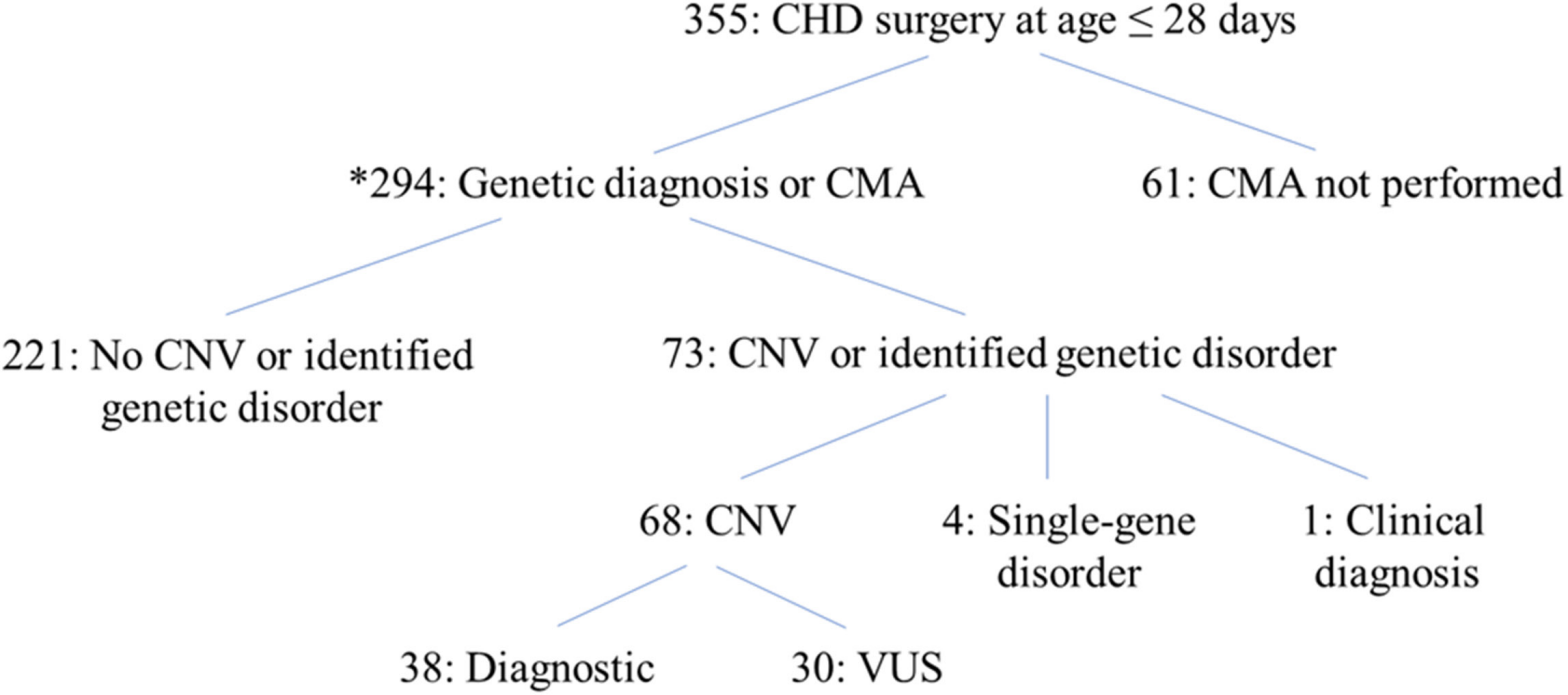
Overview of study population. Asterisk (*) indicates the 294 patients who were included for the analysis of postoperative outcomes. Genetic abnormality was defined as CNV or genetic diagnosis established by geneticist consultation. CHD indicates congenital heart disease; CMA, chromosomal microarray; CNV, copy‐number variant; and VUS, variant of uncertain significance.

Neonatal CHD surgery (aged ≤28 days) was performed in 355 patients. Clinical genetics consultation or CMA testing was performed in 311 patients (88%). Forty‐nine patients (14%) had molecular testing sent during the hospitalization in which the neonatal CHD surgery was performed (Table S1). A genetic abnormality was identified in 73 patients (21% of the cohort), and 221 without an identified genetic abnormality had a CMA confirming absence of CNV (62%) (Figure 1). Sixty‐one patients did not have a genetic abnormality or CMA sent (17%). These included 17 patients who had genetics consultation without CMA (5%) and 44 patients who did not have either consultation or CMA (12%). Taken together, implementation of the clinical algorithm was largely successful over the course of 5 years.

### Genetic Findings and Baseline Characteristics

An abnormal CNV was identified in 68 patients, including 38 with diagnostic CNV and 30 with a VUS (Table 1). Of these, the genetic diagnosis was first identified prenatally in 3 patients (22q11.2 deletion, Turner syndrome, and Recombinant 8 syndrome). Four additional patients were diagnosed with single‐gene disorders based on molecular testing sent in addition to CMA, and 1 patient was given a clinical diagnosis of a genetic syndrome (Table 2). The most frequent genetic diagnoses were 22q11.2 deletion syndrome (N=14), 15q11.2 (BP1‐BP2) deletion (N=4), Turner syndrome (N=3), 16p11.2 deletion (N=3), and CHARGE syndrome (N=3). We compared the baseline characteristics between 73 total patients with genetic abnormality and the 221 who had a negative CMA. Genetic abnormality was associated with increased frequency of prematurity and presence of ECA and lower weight at CHD surgery (Table 3). CHD surgery with CPB was less frequent in patients with genetic abnormality. Meanwhile, the STAT mortality risk categories were similar between the 2 groups; overall >70% of the operations were STAT risk 4 or 5. Taken together, comorbidities were increased in patients with genetic abnormalities, whereas the cardiac operations had similar classification of mortality risk between groups.

**Table 1:** Abnormal Copy‐Number Variants Identified in Patients Undergoing Congenital Heart Disease Surgery as Neonates (N=68 Patients)

Study ID	Syndrome/genomic disorder	Copy‐number variant	Interpretation
C06‐1036	22q11.2 deletion	(A) arr[hg19] 22q11.21(18628019‐21 704 972)x1; (B) arr[hg19] 20q13.31(55119019‐55 325 164)x3	Diagnostic
C06‐0729	22q11.2 deletion	arr[hg19] 22q11.21(18644790‐21 800 797)x1	Diagnostic
C06‐0461	22q11.2 deletion	arr[hg19] 22q11.21(18916827‐20 296 597)x1	Diagnostic
C06‐1034	22q11.2 deletion	arr[hg19] 22q11.21(18916827‐21 465 659)x1 (prenatal)	Diagnostic
C06‐0866	22q11.2 deletion	(A) arr[hg19] 22q11.2(18916827‐21 465 659)x1; (B) arr[hg19] 6q16.3(104897818‐105 337 494)x1	Diagnostic
C06‐0942	22q11.2 deletion	arr[hg19] 22q11.21(18916827‐21 465 662)x1	Diagnostic
C06‐0980	22q11.2 deletion	arr[hg19] 22q11.21(18916827‐21 465 662)x1	Diagnostic
C06‐0836*	22q11.2 deletion	arr[hg19] 22q11.21(18916827‐21 465 662)x1	Diagnostic
C06‐0325	22q11.2 deletion	(A) arr[hg19] 22q11.21(18916842‐21 465 659)x1; (B) arr[hg19] 21q22.13(38126102‐39 244 991)x3; (C) arr[hg19] 21q22.3(42842136‐48 097 372)x3	Diagnostic
C06‐0516	22q11.2 deletion	arr[hg19] 22q11.21(18916842‐21 465 659)x1	Diagnostic
C06‐0849	22q11.2 deletion	arr[hg19] 22q11.21(18916842‐20 312 661)x1	Diagnostic
C06‐0754	22q11.2 deletion	arr[hg19] 22q11.21(18916842‐21 465 662)x1	Diagnostic
C06‐0487	22q11.2 deletion	arr[hg19] 22q11.21(18916842‐21 800 797)x1	Diagnostic
C06‐0420†	22q11.2 deletion	CMA not performed. FISH: ish del(22)(q11.2q11.2)	Diagnostic
C06‐0790	15q11.2 (BP1‐BP2) deletion	(A) arr[hg19] 15q11.2(22770421–23 082 328)x1; (B) arr[hg19] 1p12(119425395‐120 527 495)x3; (C) arr[hg19] 15q14(35381603‐36 155 680)x3	Diagnostic
C06‐0622	15q11.2 (BP1‐BP2) deletion	arr[hg19] 15q11.2(22770421–23 082 328)x1	Diagnostic
C06‐0635	15q11.2 (BP1‐BP2) deletion	arr[hg19] 15q11.2(22770421–23 282 799)x1	Diagnostic
C06‐0976	15q11.2 (BP1‐BP2) deletion	arr[hg19] 15q11.2(22770421–23 282 799)x1	Diagnostic
C06‐0336	16p11.2 deletion	arr[hg19] 16p11.2(29432212‐30 191 848)x1	Diagnostic
C06‐0422	16p11.2 deletion	arr[hg19] 16p11.2(29567295‐30 178 406)x1	Diagnostic
C06‐0347	16p11.2 deletion	arr[hg19] 16p11.2(29567295‐30 226 930)x1	Diagnostic
C06‐0327	Turner	arr[hg19](X)x1; Karyotype (prenatal): 45,X	Diagnostic
C06‐0364	Turner	arr[hg19](X)x1; Karyotype: 45,X (sent together with CMA)	Diagnostic
C06‐0518	Turner (mosaic)	arr[hg19] Xp22.33q28(168546‐155 233 731)x1‐2; FISH: ish Xcen(DXZ1x2) [152]/Xcen(DXZ1x1) [48]; Karyotype: Not performed.	Diagnostic
C06‐0689	8p23.1 duplication	arr[hg19] 8p23.1(8102723‐11 945 856)x3	Diagnostic
C06‐0508	8p23.1 duplication	arr[hg19] 8p23.1(10045112‐10 971 576)x3	Diagnostic
C06‐0235	1q21.1 deletion	arr[hg19] 1q21.1q21.2(145888925‐148 665 189)x1	Diagnostic
C06‐0486	Williams	arr[hg19] 7q11.23(72643631‐74 142 190)x1	Diagnostic
C06‐0623	Recombinant 8	Formal report not available (prenatal diagnosis)	Diagnostic
C06‐0932	No	arr[hg19] 9p23p13.1(10816679‐38 771 831)x4	Diagnostic
C06‐1014	No	arr[hg19] 15q24.2q24.3(76061143‐78 201 829)x3	Diagnostic
C06‐1006	16p11.2 duplication	arr[hg19] 16p11.2(29571473‐30 177 240)x3	Diagnostic
C06‐0774	16p13.11 duplication	arr[hg19] 16p13.11p12.3(15316908‐18 172 740)x3	Diagnostic
C06‐0945	No	arr[hg19] 17p12(14087933‐15 484 858)x1	Diagnostic
C06‐0614	No	arr[hg19] 18p11.32p11.31(2255006‐4 483 453)x1	Diagnostic
C06‐0966	Alagille	arr[hg19] 20p12.3p12.1(6909491‐14 686 297)x1	Diagnostic
C06‐0417	22q11.2 duplication	arr[hg19] 22q11.21(18649166‐21 465 659)x3	Diagnostic
C06‐0650	No	arr[hg19] Xp22.21(6458939‐8 135 644)x0	Diagnostic
C06‐0592	No	arr[hg19] 1p13.2(113117899‐113 823 881)x3	VUS
C06‐0989	No	arr[hg19] 1p36.32(2694431‐3 202 884)x3	VUS
C06‐0371	No	arr[hg19] 1q44(247824375‐248 795 277)x1	VUS
C06‐0859	No	arr[hg19] 2q36.3(230590607‐230 702 741)x3	VUS
C06‐0598	No	arr[hg19] 3q26.31(173860979‐174 420 418)x3	VUS
C06‐0776	No	arr[hg19] 4q22.1(92787339‐93 628 151)x3	VUS
C06‐0608	No	arr[hg19] 5q35.3(179085550‐179 649 205)x3	VUS
C06‐0826	No	arr[hg19] 6q13(71263529‐72 470 456)x3	VUS
C06‐0552	No	arr[hg19] 7q11.2(69517881‐69 971 310)x1	VUS
C06‐0482	No	arr[hg19] 7q33(134114282‐134 445 433)x1	VUS
C06‐0702	No	arr[hg19] 7q36.2(153524141‐153 763 852)x3	VUS
C06‐0470	No	arr[hg19] 9q21.11(71599176‐71 849 431)x3	VUS
C06‐0937	No	arr[hg19] 10q21.1(53831082‐53 990 160)x3	VUS
C06‐0935	No	arr[hg19] 10q21.3(68249298‐68 414 336)x1	VUS
C06‐0835	No	arr[hg19] 10q26.3(134290543‐135 167 541)x3	VUS
C06‐0604	No	arr[hg19] 11p13(32969977‐33 561 504)x3	VUS
C06‐0753	No	arr[hg19] 11q22.1(99610283‐100 681 379)x3	VUS
C06‐0286	No	arr[hg19] 11q22.1.1q25(100321535‐132 291 355)x3	VUS
C06‐0760	No	arr[hg19] 13q33.1q33.2(104740013‐106 177 489)x1	VUS
C06‐0645	No	arr[hg19] 15q13.2q13.3(31073668‐32 439 524)x3	VUS
C06‐0648	No	arr[hg19] 17p13.3(1092257‐1 227 927)x3	VUS
C06‐0690	No	arr[hg19] 17q25.1(71834171‐72 681 118)x3	VUS
C06‐0520	No	arr[hg19] 18p11.21(13424988‐13 903 119)x3	VUS
C06‐0867	No	arr[hg19] 19q13.33(50042015‐50 612 095)x1	VUS
C06‐1035	No	arr[hg19] 20p12.2(9751678‐9 968 858)x3	VUS
C06‐0416	No	arr[hg19] 21q22.3(46055326‐46 530 170)x3	VUS
C06‐0580	No	arr[hg19] 22q12.2(29833216‐30 000 917)x4	VUS
C06‐0825	No	arr[hg19] 22q12.3(36614296‐36 708 483)x3	VUS
C06‐0588	No	(A) arr[hg19] Xq27.1(138377872‐139 000 629)x2; (B) arr[hg19] 20q13.12(45973939‐46 209 563)x3	VUS
C06‐0978	No	arr[hg19] Xq28(154296581‐154 325 127)x0	VUS

CMA indicates chromosomal microarray; FISH, fluorescence in situ hybridization; ID, identification; and VUS, variant of uncertain significance.

* Also found homozygosity of 24.1% of the genome consistent with parental consanguinity.

^†^ Also carries pathogenic variant in *PAX6*.

**Table 2:** Single‐Gene Disorders Identified in Patients Undergoing Neonatal Congenital Heart Disease Surgery (N=5)

Study ID	Syndrome	Molecular finding	CMA result	CMA interpretation
C06‐0541	CHARGE	Likely pathogenic variant in *CHD7*: c.3226_3227delAA	Normal	Normal
C06‐0770	CHARGE	Pathogenic variant in *CHD7*: c.678_680delTATinsAA	Normal	Normal
C06‐0931	CHARGE	Pathogenic variant in *CHD7*: c.6070C > T, p.R2024X	Normal	Normal
C06‐0984	SMA type 1	*SMN1* (−/−)	arr[hg19] Xq27.1(138808850‐139 338 275)x2	VUS
C06‐0267	Kabuki*	None performed	Normal	Normal

CMA indicates chromosomal microarray; ID, identification; SMA, spinal muscular atrophy; and VUS, variant of uncertain significance.

* Clinical diagnosis.

**Table 3:** Baseline Characteristics in 355 Patients Who Underwent Neonatal Surgery for CHD

	Total, N=294	Genetic abnormality, N=73	No CNV, N=221	*P* value*	NA, N=61
Sex				0.937†	
Boys	170 (57.8%)	43 (58.9%)	127 (57.5%)		43 (70.5%)
Girls	124 (42.2%)	30 (41.1%)	94 (42.5%)		18 (29.5%)
Race				0.375‡	
White	248 (84.4%)	60 (82.2%)	188 (85.1%)		52 (85.2%)
Black	31 (10.5%)	11 (15.1%)	20 (9.0%)		7 (11.5%)
Other	11 (3.7%)	2 (2.7%)	9 (4.1%)		2 (3.3%)
Unknown	4 (1.4%)	0 (0.0%)	4 (1.8%)		0 (0.0%)
Prematurity, GA <37 wk				0.009†	
Yes	56 (19.0%)	22 (30.1%)	34 (15.4%)		9 (14.8%)
No	238 (81.0%)	51 (69.9%)	187 (84.6%)		52 (85.2%)
Extracardiac anomaly				0.034†	
Yes	120 (40.8%)	38 (52.1%)	82 (37.1%)		11 (18.0%)
No	174 (59.2%)	35 (47.9%)	139 (62.9%)		50 (82.0%)
Weight at CHD surgery, kg				0.009	
Mean (SD)	3.17 (0.67)	2.99 (0.65)	3.23 (0.67)		3.23 (0.56)
Intubated prior to CHD surgery				0.719†	
Yes	66 (22.4%)	18 (24.7%)	48 (21.7%)		9 (14.8%)
No	228 (77.6%)	55 (75.3%)	173 (78.3%)		52 (85.2%)
Cardiopulmonary bypass				0.048†	
Yes	132 (44.9%)	25 (34.2%)	107 (48.4%)		31 (50.8%)
No	162 (55.1%)	48 (65.8%)	114 (51.6%)		30 (49.2%)
STAT mortality risk category				>0.999†	
1, 2, or 3	83 (28.2%)	21 (28.8%)	62 (28.1%)		23 (37.7%)
4 or 5	211 (71.8%)	52 (71.2%)	159 (71.9%)		38 (62.3%)

In the column headed NA (Not analyzed) were patients without genetic diagnosis or CMA testing who were not included in the statistical analysis. CHD indicates congenital heart disease; CNV, copy‐number variant; GA, gestational age; Other, Includes 9 Asian; 2 Native American; 2 Other, not otherwise specified; and STAT, Society of Thoracic Surgeons‐European Association for Cardio‐Thoracic Surgery.

* *P* value from 2‐sample *t* test for continuous variables and from Pearson χ^2^ test or Fisher exact test for categorical variables.

^†^ Pearson χ^2^ test was applied.

^‡^ Fisher exact test was applied.

### Postoperative Outcomes

Genetic abnormality was not associated with longer duration of postoperative hospitalization, requirement for intubation for >7 days postoperatively, or need for extracorporeal membrane oxygenation (ECMO) postoperatively (Table 4). Of note, patients with ECA were significantly less likely to receive postoperative ECMO (15 of 174 without ECA versus 2 of 120 with ECA) (odds ratio [OR], 0.18 [95% CI, 0.02–0.80]; *P*=0.011 using the Fisher exact test). The operative mortality rate in patients with genetic abnormality was approximately 2 times higher than for those without genetic abnormality (9.6% versus 4.1%), although this difference was not statistically significant (*P*=0.080 using the Fisher exact test). The genetic abnormalities that were identified in mortality cases are shown in Table 5. These included 4 patients diagnosed with well‐characterized genetic syndromes and 3 with CNVs that were classified as VUS. The mortality rate of patients with diagnostic genetic evaluation (4/43=9.3%) was similar to patients with VUS on CMA (3/30=10.0%), although the surgeries in the VUS group were less commonly on CPB and had fewer STAT risk 4 or 5 operations (Table 6). Notably, the only 2 patients with 22q11.2 deletion who had operative mortality had an additional CMA finding (Table 5), and 13 of the 14 patients with 22q11.2 deletion had CMA completed (Table 1). One of these patients had 2 duplications on chromosome 21. In the other patient, ROH constituted approximately 24% of the genome consistent with parental consanguinity, increasing the likelihood for ≥1 co‐occurring recessive disease(s). Among patients who underwent higher risk operations (STAT 4 or 5), the operative mortality rate was 13.5% in patients with genetic abnormality (7/52 patients) versus 5.0% of patients without genetic abnormality (8/159 patients) (*P*=0.058 using the Fisher exact test). Patients who had either genetic abnormality or ECA had increased operative mortality (13/155, 8.4%) compared with those without such findings (3/139, 2.2%) (*P*=0.021 using the Fisher exact test). Genetic abnormality was not independently associated with poorer outcomes, although there was a trend for increased operative mortality, particularly in the operations that have higher mortality risk at baseline.

**Table 4:** Comparison of Postoperative Outcomes Between Patients With Genetic Abnormality Versus Normal Genetic Evaluation That Included a Negative Chromosomal Microarray

	Total, N=294	Genetic abnormality, N=73	No CNV, N=221	*P* value*	NA, N=61
Days of hospitalization after CHD surgery				0.887	
Mean (SD)	36.0 (43.2)	36.7 (42.3)	35.8 (43.6)		28.3 (28.5)
Intubation >7 d postoperatively, N (%)				0.575†	
Yes	82 (27.9%)	18 (24.7%)	64 (29.0%)		17 (27.9%)
No	212 (72.1%)	55 (75.3%)	157 (71.0%)		44 (72.1%)
ECMO postoperatively, N (%)				0.577‡	
Yes	17 (5.8%)	3 (4.1%)	14 (6.3%)		6 (9.8%)
No	277 (94.2%)	70 (95.9%)	207 (93.7%)		55 (90.2%)
Operative mortality, N (%)				0.080‡	
Yes	16 (5.4%)	7 (9.6%)	9 (4.1%)		1 (1.6%)
No	278 (94.6%)	66 (90.4%)	212 (95.9%)		60 (98.4%)

In the column headed NA (Not analyzed) were patients without genetic diagnosis or chromosomal microarray testing who were not included in the statistical analysis. CNV indicates copy‐number variant; and ECMO, extracorporeal membrane oxygenation.

* *P* value from 2‐sample *t* test for continuous variables and from χ^2^ test or Fisher exact test for categorical variables.

^†^ Pearson χ2 test was applied.

^‡^ Fisher exact test was applied.

**Table 5:** Genetic Abnormalities in Patients With Operative Mortality Following Neonatal Congenital Heart Disease Surgery

Genetic abnormality	Cytogenetic finding	Extracardiac anomaly	Primary procedure	STAT category
Williams syndrome	7q11.23 deletion	No	TAPVR repair	4
CHARGE syndrome	Normal CMA	Yes	Shunt, systemic to pulmonary, central (shunt from aorta)	4
22q11.2 deletion syndrome	22q11.2 deletion and 24.1% ROH	No	TOF, absent pulmonary valve repair	4
22q11.2 deletion syndrome	22q11.2 deletion, 21q22.13 duplication, and 21q22.3 duplication	Yes	PA banding	4
VUS	15q13.2‐q13.3 duplication	Yes	Hybrid approach stage 1, application of RPA and LPA bands	5
VUS	22q12.2 triplication	No	Hybrid approach stage 1, application of RPA and LPA bands	5
VUS	Xq28 deletion	No	Shunt, systemic to pulmonary, modified Blalock‐Taussig shunt	4

CMA indicates chromosomal microarray; LPA, left pulmonary artery; PA, pulmonary artery; ROH, region of homozygosity; RPA, right pulmonary artery; STAT, Society of Thoracic Surgeons‐European Association for Cardio‐Thoracic Surgery; TAPVR, total anomalous pulmonary venous return; TOF, tetralogy of Fallot; and VUS, variant of uncertain significance.

**Table 6:** Baseline Characteristics and Outcomes Between Genetic Testing Groups

	Diagnostic, N=43	CMA‐VUS, N=30	CMA‐ROH, N=9	No CNV or ROH, N=212	NA, N=61
Baseline characteristics					
Male sex	56%	63%	67%	57%	70%
White race	81%	83%	78%	87%	85%
Premature, gestational age <37 wk	33%	27%	11%	16%	15%
Extracardiac anomaly present	53%	50%	33%	37%	18%
Weight at time of CHD surgery, kg, mean (SD)	2.96 (0.62)	3.04 (0.70)	3.10 (0.35)	3.23 (0.68)	3.23 (0.56)
Intubated prior to CHD surgery	23%	27%	11%	22%	15%
Cardiopulmonary bypass surgery	37%	30%	33%	49%	51%
STAT mortality risk category 4 or 5	77%	63%	89%	71%	62%
Postoperative outcome					
Days of hospitalization after CHD surgery, mean (SD)	38.9 (42.7)	27.3 (35.0)	24.9 (24.8)	34.2 (40.3)	27.8 (28.7)
Intubation >7 d postoperatively	26%	23%	11%	30%	28%
ECMO postoperatively	5%	3%	0	7%	10%
Mortality	9.3%	10.0%	0	4.3%	1.6%

CHD indicates congenital heart disease; CMA, chromosomal microarray; CNV, copy‐number variant; ECMO, extracorporeal membrane oxygenation; NA, CMA not completed; ROH, region(s) of homozygosity; STAT, Society of Thoracic Surgeons‐European Association for Cardio‐Thoracic Surgery; and VUS, variant of uncertain significance.

### Analysis of Patients Without Extracardiac Anomalies

**Figure 2:**
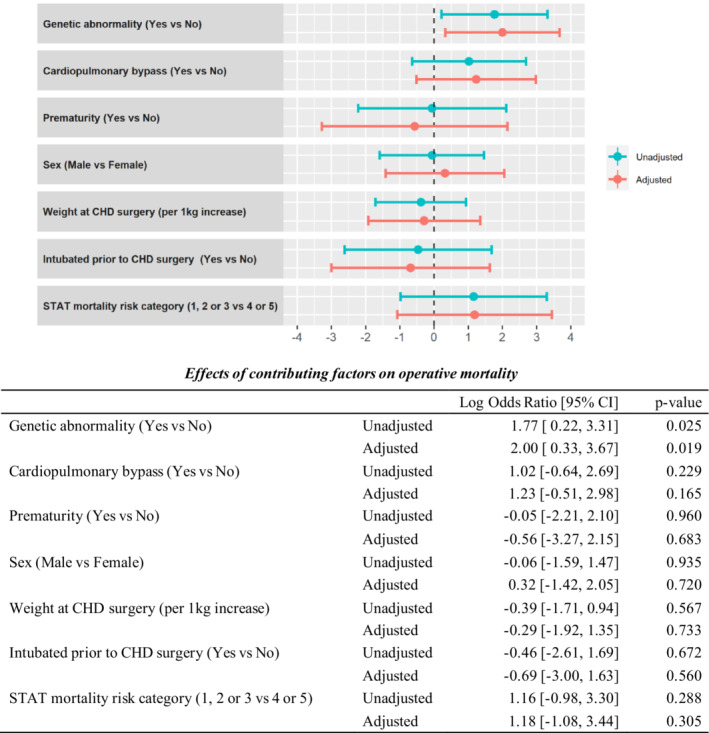
Results of multivariate analysis for characteristics associated with operative mortality among neonates without extracardiac anomaly. Log odds ratios depicted on the *x* axis are estimated through multivariate logistic regression model. CHD indicates congenital heart disease; and STAT, Society of Thoracic Surgeons‐European Association for Cardio‐Thoracic Surgery.

The presence of ECAs has been previously associated with poorer surgical outcomes.^7^ In the present study, patients with genetic abnormality were more likely to have an ECA (Table 3). To determine the impact of genetic abnormality independent of ECA, the cohort was subclassified according to the presence or absence of ECAs. Genetic abnormality was identified in 20.1% of the 174 patients without ECAs (Table 7). In patients without ECAs, a genetic abnormality was associated with prematurity and lower weight at CHD surgery, whereas CPB and STAT categories did not significantly differ (Table S2). Genetic abnormality was not associated with postoperative length of hospitalization, need for postoperative ECMO, or intubation for >7 days postoperatively (Table 7). Meanwhile, genetic abnormality was significantly associated with increased operative mortality (11.4% versus 2.2%) (*P*=0.031 using the Fisher exact test). A multivariate analysis for operative mortality was performed, including genetic abnormality, CPB, STAT mortality risk category (1, 2, or 3 versus 4 or 5), prematurity, sex, weight at surgery, and whether intubated prior to surgery. In this model, a genetic abnormality was independently associated with operative mortality (log OR, 2.0 [95% CI, 0.33–3.67]; *P*=0.019) (Figure 2). In contrast to these findings, among the 120 patients who had an ECA, the operative mortality rate was similar between those with genetic abnormality (3/38 patients, 7.9%) and without genetic abnormality (6/82 patients, 7.3%). This result suggests that in patients with ECA, the interrelationship between ECA and genetic abnormality likely complicates the ability to discriminate their relative effects on mortality. Meanwhile, the findings provide evidence that genetic abnormality significantly impacts outcome in the absence of ECAs. This strengthens the rationale for performing genetics evaluation in all patients with severe CHD regardless of ECA status.

**Table 7:** Postoperative Outcomes in Patients Without an Extracardiac Anomaly

	Total, N=174	Genetic abnormality, N=35	No CNV, N=139	*P* value*	NA, N=50
Days of hospitalization after CHD surgery				0.583	
Mean (SD)	32.7 (40.2)	36.2 (42.8)	31.8 (39.7)		23.7 (20.2)
Intubation >7 d postoperatively				>0.999†	
Yes	42 (24.1%)	8 (22.9%)	34 (24.5%)		14 (28.0%)
No	132 (75.9%)	27 (77.1%)	105 (75.5%)		36 (72.0%)
Operative mortality				0.031‡	
Yes	7 (4.0%)	4 (11.4%)	3 (2.2%)		1 (2.0%)
No	167 (96.0%)	31 (88.6%)	136 (97.8%)		49 (98.0%)
ECMO postoperatively				>0.999‡	
Yes	15 (8.6%)	3 (8.6%)	12 (8.6%)		5 (10.0%)
No	159 (91.4%)	32 (91.4%)	127 (91.4%)		45 (90.0%)

In the column headed NA (Not analyzed) were patients without genetic diagnosis or chromosomal microarray testing who were not included in the statistical analysis. CHD indicates congenital heart disease; CNV, copy‐number variant; and ECMO, extracorporeal membrane oxygenation.

* *P* value from 2‐sample *t* test for continuous variables and from χ^2^ test or Fisher exact test for categorical variables.

^†^ Pearson χ^2^ test was applied.

^‡^ Fisher exact test was applied.

## DISCUSSION

This study was performed in a pediatric cardiac center where CVG evaluations were performed systematically over a 5‐year period. Genetic abnormalities were identified frequently in neonates undergoing CHD surgery. Patients with diagnostic genetic findings or VUS on CMA had similar increases in the rates of operative mortality. CMA identified genetic abnormalities that likely would not have been otherwise detected and were associated with mortality. These included CNVs in patients with apparently isolated CHD and additional CMA findings in both patients with 22q11.2 deletion who did not survive. We did not identify significant associations between genetic abnormality and the secondary postoperative outcomes that were measured, including length of stay, need for ECMO, or prolonged intubation.

### With Increased Identification of Genetic Abnormalities in CHD, There Is A Need to Understand Their Clinical Impact in the Context of Systematic Evaluation

The current study demonstrates that clinical CVG algorithms have high yields. The observed rate of genetic abnormality in the present study is similar to results from 2 different centers where similar algorithms were successfully implemented by CVG providers.^12^, ^13^ Prior studies have investigated the association between genetic abnormalities and postoperative survival during earlier eras when CMA testing was infrequently performed.^7^, ^14^, ^15^, ^16^, ^17^, ^18^, ^19^, ^20^ The overall conclusion from these studies was that, with some exceptions, genetic diagnoses generally increased the risk for poorer outcomes including mortality and other secondary outcomes. However, the interpretation of this literature was complicated by inconsistency in genetics evaluations within study cohorts; variable definitions of what constitutes a genetic abnormality (sometimes including ECA without a specific genetic diagnosis); accuracy of genetic data collection, which can be fraught because of the inherent complexity of genetic testing and interpretation; and lack of documentation of what testing or evaluations were performed, including whether normal cases had negative testing. Since then, there has emerged more advanced genetic testing, increased understanding of genetic causes of CHD, and clinical initiatives to implement more comprehensive genetics evaluations.^21^

Several recent studies have used broad genetic testing technologies to investigate the clinical impact of genetic abnormalities in CHD and compared survival among patients who underwent the same type of genetic testing. These studies are summarized along with their main findings in Table 8.^22^, ^23^, ^24^ For the present study, our center's concerted and sustained clinical initiative to perform CVG evaluation and genetic testing of critically ill infants with CHD has helped to minimize some of the challenges faced by these prior studies. First, our CVG service was well integrated within the intensive care unit, and clinical geneticist consultations and testing were performed soon after birth. This decreased the likelihood of missed genetic diagnoses in operative mortality cases, minimizing survival bias. Only 1 mortality case did not receive CVG consultation or testing. The testing performed in this study was clinical and a routine practice, and therefore did not face obstacles inherent to research enrollment and testing, which likely increased the ascertainment of eligible patients. Importantly, we can report not only the number of patients who were excluded for not having CMA, which was relatively infrequent, but also describe their characteristics. Our systematic clinical approach to CVG evaluations and testing likely decreased selection bias, while also presenting a real‐world application of genetic screening and risk assessment in the clinical setting.

**Table 8:** Summary of Studies That Have Investigated the Impact of Genetic Abnormality on Survival in CHD Using Broad Testing

Study characteristics	Kim, 2016	Dailey‐Schwartz, 2018	Boskovski, 2020	Current study
Research vs clinical	Research	Clinical	Research	Clinical
Location	Single center	Single center	Multicenter	Single center
Study years	Enrollment 1998 to 2003	2008 to 2016	Enrollment 2010 to 2016	2015 to 2020
CHD types	Any	HLHS only	Any	Any
Age at surgery	CPB surgery ≤6 mo	Presumed neonates	Any (eg, 35.0% neonates, 4.4% adults)	Age ≤28 d
Genetic selection criteria	Excluded if genetic or phenotypic evidence of a syndrome was known at enrollment or if was later identified by research testing; excluded if multiple congenital anomalies present	None specified	Excluded patients with pre‐enrollment genetic diagnosis; included patients with genetic diagnosis identified in research testing; excluded patients with a known first degree relative with CHD	Excluded patients with trisomy 21 or trisomy 18
No. of cases included in primary analysis of survival	422	105	1268	294
No. of cases potentially eligible that were not included in primary analysis	253 (number includes 55 enrolled cases found to have DiGeorge syndrome or other chromosomal/genetic abnormality)	Not specified	Not specified	61
Genetic testing	Research CMA	Clinical CMA	Research exome sequencing	Clinical CMA or other testing as indicated
Geneticist examination	Yes	Not specified	Not a part of study	Yes
Criteria for abnormal CNV	Interval size >300 kb and overlapped any gene(s) and did not have >50% overlap with a nonpathogenic CNV present in Database of Genomic Variants	Clinical interpretation: known disorder or VUS	De novo and present in <1% of cohort and spanning ≥3 exons	Clinical interpretation: diagnostic or VUS
Criteria for abnormal sequence variant	Not analyzed	Not analyzed	De novo and predicted to be deleterious on protein expression/function	Clinical interpretation: pathogenic or likely pathogenic
No. of cases with genetic abnormality	51 (12.1%)	Known genetic disorder: 9 (8.6%); VUS: 26 (24.7%)	143 (CNV or sequence variant) (11.3%)	73 (24.8%), including diagnostic (N=43, 14.6%) or VUS (N=30, 10.5%)
Duration of follow‐up	≥2.5 y after initial operation	Not specified	Median 2.65 y after initial operation	During hospitalization or within 30 d of initial operation
Primary survival analysis	Decreased transplant‐free survival	Decreased 1‐y transplant‐free survival for known genetic disorder but not for VUS	Decreased transplant‐free survival	No statistical difference in survival
Survival analysis of patients without ECA	Decreased (equivalent to primary analysis because excluded patients with multiple congenital anomalies)	Not assessed	Decreased transplant‐free survival	Decreased survival

CHD indicates congenital heart disease; CMA, chromosomal microarray; CNV, copy‐number variant; CPB indicates cardiopulmonary bypass; ECA, extracardiac anomaly; HLHS, hypoplastic left heart syndrome; and VUS, variant of uncertain significance.

### Cardiac Considerations for Study Inclusion and Early Versus Late Survival Outcomes

CHD is morphologically and physiologically heterogeneous, and the timing, frequency, and complexity of surgical interventions variable. The studies included in Table 8 selected the types of CHDs to varying degrees. The sole cardiac criterion for eligibility in the present study was requirement of neonatal CHD surgery. We analyzed outcomes that occurred during the hospitalization when the initial CHD surgery took place. Because the focus was on early postoperative survival, the impact of the type of operation on outcome was controlled using the STAT category in multivariable analysis and by performing subanalysis restricted to STAT category 4 or 5 operations. The statistical rationale for using cohorts with heterogeneous types of CHD is that adequate sample sizes are needed to analyze the impact of rare genetic abnormalities. Also, the known genetic causes of CHD have variable expressivity and present with a range of different types of CHD. Imposing stringent criteria for types of CHD in the study of outcomes may decrease the ability to identify the risks that are associated with rare genetic diagnoses that have variable CHD presentations.

There are conceptual differences for analyzing the impact of genetic abnormalities on early postoperative survival, such as in the present study, versus their impact on longitudinal survival in other studies. In the early postoperative period, factors that decrease cardiorespiratory reserve or alter fluid balance regulation via involvement of renal or lymphatic systems are critically important. Patients are closely monitored for infection, neurological changes such as seizures, and arrhythmia. In the ambulatory setting, development of these complications may be more difficult to identify and treat promptly. Congenital aerodigestive anomalies that are successfully repaired or palliated to achieve hospital discharge may have risk for later complications and chronic comorbidity. Increased pulmonary artery pressure secondary to chronic respiratory insufficiency or vascular anomalies (congenital or maladaptive) can negatively impact longitudinal outcomes, particularly in palliated single‐ventricle physiologies that depend on passive pulmonary blood flow. Thus, genetic abnormalities may differentially impact acute or longitudinal outcomes, depending on the associated phenotypes and systems involved.

### Genetic Considerations for Study Inclusion

The current study includes all patients with an identified genetic diagnosis except for trisomy 21 or 18. Syndromic conditions, such as 22q11.2 deletion and CHARGE syndrome, were included to understand the overall impact of genetic abnormalities that were identified via our center's inpatient CVG algorithm. We excluded trisomy 21 because, unlike other genetic syndromes with strong CHD association, trisomy 21 is routinely diagnosed during standard prenatal and postnatal care, large studies have demonstrated favorable outcomes, and trisomy 21 is more common than other rare genomic disorders.^25^, ^26^, ^27^ Trisomy 18 was excluded because of the known high lethality that is largely independent of CHD.

### CNVs Classified as VUSs May Impact Clinical Outcomes in CHD

The clinical testing and interpretations in our study were performed by CVG experts, and abnormal CMA results included diagnostic variants and VUSs. The similar mortality rates between patients with diagnostic results and VUSs suggest that some VUSs may impact early postoperative survival. From their specified criteria, it is likely that several CNVs included as abnormal in the studies of Kim et al and Boskovski et al (Table 8) would be classified as VUSs clinically. These results together raise an interesting possibility that genetic variants may not need to be independently or definitively causative of CHD to be clinically impactful.

Previous studies in 22q11.2 deletion syndrome have predominantly determined that overall postoperative mortality is not greatly increased.^17^, ^18^, ^28^, ^29^, ^30^, ^31^ In the current study, we found that among 14 cases with 22q11.2 deletion, the only 2 operative mortalities occurred in cases who also had additional CMA findings. These data, albeit small in numbers, lead to a hypothesis that additional genetic abnormalities significantly impact survival in 22q11.2 deletion. Genome‐wide testing with CMA may not only help to identify pathogenic CNVs at greater resolution than traditional fluorescence in situ hybridization (FISH),^5^ but also may identify such additional variants that are clinically important postoperatively. Larger data sets to test this hypothesis may become available as more clinical centers adopt routine CMA testing.

### Genetic Abnormalities Are Common in Apparently Isolated CHD Cases and Are Clinically Significant

As expected, genetic abnormalities were more frequent in patients with ECAs. However, 20% of patients without an ECA were found to have a genetic abnormality, which is comparable to prior observations in a similarly evaluated cohort.^12^ In the current study, a genetic abnormality was associated with increased mortality in patients without ECA. Kim et al excluded patients with multiple ECAs, and found that CNVs were associated with decreased survival. Similarly, the genetic abnormalities defined in Boskovski et al were associated with decreased survival in patients who did not have ECAs. These data, taken together, including the current results acquired from a strictly clinical setting, indicate that routinely testing patients with critical CHD clinically for CNVs can identify increased risk for poor outcomes, including when a genetic diagnosis may not be otherwise suspected.

### Most Patients With Pathogenic CNVs Survived Neonatal CHD Surgery

Although association with increased mortality was observed, the majority of patients with genetic abnormality, including pathogenic CNVs, survived neonatal CHD surgery. Survivors included all 3 patients with Turner syndrome, all 3 with 16p11.2 deletion, and both with 8p23.1 duplication. Boskovski et al identified 15q11.2 deletion as having increased mortality risk.^24^ In the present study, all 4 neonates with 15q11.2 deletion survived, including 2 with STAT risk category 5 procedures (both Norwood operations) and 1 with STAT risk category 4 (total anomalous pulmonary venous return repair). A 15q11.2 deletion may be an example of a condition that can be successfully managed postoperatively but may predispose to complications that decrease longer‐term survival. Another pertinent finding in this study was that the duration of hospitalization, need for ECMO, and prolonged postoperative intubation were not increased in patients with genetic abnormality. It is possible that the specific type of CHD or operation (eg, Norwood procedure) has a stronger effect on these outcomes than on mortality. Analysis of these outcomes may also be confounded by mortalities that occur shortly after an operation.

The limitations of this study include that it was retrospective and single center. Overall, the mortality rate was low, which limited statistical power. Requiring CNVs to be ruled out with CMA, the early postnatal timing of evaluations, and the standardized approach to evaluation increase specificity and clinical validity. A minority of patients were excluded from the primary analyses because CMA was not completed. Approximately 50% of these excluded patients had surgery in 2015, which was early in the implementation of the CVG clinical algorithm. By the years 2018 to 2020, only 5 of the 150 (3.3%) eligible cases did not have CMA. Thus, the integration of routine CVG evaluation into pediatric cardiac critical care settings took a short period of time and was successful. This study was clinical and did not include research‐based genetic testing. Parental testing for CNVs that were classified as VUSs was pursued only when thought to be highly informative. The ability to complete parental testing for VUSs during the patient's initial hospitalization was limited by practical factors such as parental sample availability, as well as uninsured financial costs. The indications and feasibility of parental testing or additional genetic testing are often reconsidered in outpatient CVG follow‐up. This report ascertained all neonates who underwent surgery and does not include cases where comfort care was chosen or were unable to be stabilized preoperatively, which were rare. The sample size of our study was relatively small, which may lead to relatively wide confidence intervals for a few covariates (eg, STAT). We further estimated the variance inflation factors for all covariates in the multiple logistic regression model. The values ranged between 1.03 and 1.45 and did not suggest collinearity among covariates.

In conclusion, this study provides a novel perspective of the impact of genetic abnormalities on early postoperative outcomes when CVG consultations and genetic testing are systematically performed. The first clinical implication of this study is that CVG evaluations of neonates with CHD have demonstrable yield and identify genetic abnormalities that would not otherwise be suspected and should be routine. Second, genetic abnormalities in patients without ECA or additional genetic findings in patients with 22q11.2 deletion may have increased risk for postoperative mortality, and adjusting clinical management accordingly may improve outcomes. The consensus statement recommending CMA as a first‐tier test in patients who have congenital anomalies was first published in 2010.^32^ It has required time and resources to incorporate these guidelines faithfully into the clinical care of infants with critical CHD. Given recent evidence supporting the use of whole genome sequencing in critically ill neonates,^33^ we anticipate that new guidelines may be on the near horizon that would apply to infants with critical CHD. In this article, we have discussed the challenges associated with the study of genetic abnormalities and outcomes in CHD, which apply to research and clinical cohorts. Understanding the nature and depth of clinical genetic evaluation and testing that was completed for all included patients is of paramount importance, if not a prerequisite, for studies that seek to determine the clinical impact of genetic abnormalities on outcomes in CHD. Concerted clinical efforts and collaboration to adopt genomic testing practices quickly in a standardized manner between centers may be the most efficient path toward understanding the clinical impact of genetic abnormalities in CHD. By learning to crawl in this manner we will develop the fundamental basis to walk and run.

## Sources of Funding

This work was supported by the American Heart Association Transformational Award AHA 19TPA34850054 (S.M.W.), National Institutes of Health T35 HL110854 (M.H.), and National Institutes of Health K23 HL141667 (B.J.L.).

## Disclosures

None.

## Supporting information

Tables S1–S2
