# Supplementary material for: Learning to Crawl: Determining the Role of Genetic Abnormalities on Postoperative Outcomes in Congenital Heart Disease
Source: J Am Heart Assoc. 2022 Sep 29;11(19):e026369. doi: 10.1161/JAHA.122.026369 (PMC9673727; doi:10.1161/JAHA.122.026369)
Supplement: Supplementary file 1 — Tables S1–S2 [file JAH3-11-e026369-s001.pdf]

Supplemental Material

Table S1. Types and frequencies of molecular genetic testing sent during the hospitalization in which the neonatal CHD surgery was performed (N=355).

Test	Number of patients
Exome-based congenital anomalies panel	27
Heterotaxy panel	8
Noonan/RASopathy panel	7*
Exome sequencing	5
Single-gene: *CHD7*	2
Single-gene: *SMN1*	1*
Total	49**

*Counts include one patient who had both Noonan/RASopathy panel and *SMN1* single-gene testing.

**Forty-eight of 49 patients also had chromosomal microarray completed.

Table S2. Baseline characteristics in neonatal CHD cases without an extracardiac anomaly.

	Total (N=174)	Genetic abnormality (N=35)	Normal (N=139)	p-value*	NA (N=50)
Sex				0.247[P]	
Male	102 (58.6%)	17 (48.6%)	85 (61.2%)		34 (68.0%)
Female	72 (41.4%)	18 (51.4%)	54 (38.8%)		16 (32.0%)
Race				0.231[F]	
White	152 (87.4%)	29 (82.9%)	123 (88.5%)		42 (84.0%)
Black	13 (7.5%)	5 (14.3%)	8 (5.8%)		6 (12.0%)
Other	6 (3.4%)	1 (2.9%)	5 (3.6%)		2 (4.0%)
Unknown	3 (1.7%)	0 (0.0%)	3 (2.2%)		0 (0.0%)
Prematurity (GA < 37 weeks)				0.024[P]	
Yes	26 (14.9%)	10 (28.6%)	16 (11.5%)		7 (14.0%)
No	148 (85.1%)	25 (71.4%)	123 (88.5%)		43 (86.0%)
Weight at CHD surgery				0.006	
Mean (SD)	3.23 (0.68)	2.95 (0.64)	3.3 (0.67)		3.24 (0.56)
Intubated prior to CHD surgery				>0.999[P]	
Yes	36 (20.7%)	7 (20.0%)	29 (20.9%)		6 (12.0%)
No	138 (79.3%)	28 (80.0%)	110 (79.1%)		44 (88.0%)
Cardiopulmonary bypass				0.199[P]	
Yes	84 (48.3%)	13 (37.1%)	71 (51.1%)		25 (50.0%)
No	90 (51.7%)	22 (62.9%)	68 (48.9%)		25 (50.0%)
STAT mortality risk category				0.801[P]	
1,2, or 3	59 (33.9%)	13 (37.1%)	46 (33.1%)		20 (40.0%)
4 or 5	115 (66.1%)	22 (62.9%)	93 (66.9%)		30 (60.0%)

*P-value from two-sample t-test for continuous variables and from Pearson's Chi-square test or Fisher's exact test for categorical variables. [P] Pearson's Chi-square test was applied; [F] Fisher's exact test was applied.

The column headed NA were patients without genetic diagnosis or CMA testing and were not included in the statistical analysis.

CNV: copy-number variant; GA: gestational age; SD: standard deviation
